# Spinal dural arteriovenous fistula: A rare but treatable disease that should not be missed by orthopedic surgeons

**DOI:** 10.3389/fneur.2022.938342

**Published:** 2022-09-20

**Authors:** Baohui Yang, Teng Lu, Xijing He, Haopeng Li

**Affiliations:** Department of Orthopedics, Second Affiliated Hospital of Xi'an Jiaotong University, Xi'an, China

**Keywords:** spinal dural arteriovenous fistula, misdiagnosis, spinal angiography, microsurgery resection, MDT

## Abstract

**Objective:**

Spinal dural arteriovenous fistula (SDAVF) is a rare disease that is often misdiagnosed by orthopedic surgeons. We analyzed the reasons for the misdiagnosis and proposed countermeasures.

**Methods:**

Twenty-two SDAVF patients who were initially treated in orthopedics were included. The patients were divided into a correct diagnosis group (A) and a misdiagnosis group (B). The clinical data and prognosis were evaluated.

**Results:**

There were 10 patients in group A and 12 patients in group B. The clinical manifestations included limb numbness, weakness, and bladder and bowel dysfunction. Among these patients without spinal degenerative diseases which had typical magnetic resonance imaging (MRI) features in Group A were more than Group B (*P* < 0.05). More patients had spinal degenerative diseases in group B. In group A, seven patients were primarily diagnosed with a SDAVF after multidisciplinary teamwork (MDT). In group B, five patients were misdiagnosed with lumbar spinal stenosis, four with lumbar disc herniation, two with thoracic spinal stenosis, and one with cervical spinal stenosis and lumbar spinal stenosis and underwent cervical spinal canal and lumbar spinal canal decompression. The length of time for confirming the diagnosis was 7 months longer in group B than in group A. All patients underwent microsurgery treatment. The average follow-up duration was 11 months. The modified Aminoff-Logue Disability Scale scores showed a statistically significant difference in improvement between the two groups (*P* < 0.05).

**Conclusion:**

when patients with dysuria especially, have intermittent spinal nerve dysfunction, the possibility of SDAVF should be considered. Awareness of the specific clinical and spinal cord edema and flow voids on MRI of a SDAVF needs to be promoted for orthopedic surgeons. Timely MDT is an important measure for reducing misdiagnosis, and steroids or inappropriate surgery should be avoided until a SDAVF is completely excluded.

## Introduction

Spinal dural arteriovenous fistula (SDAVF) is a relatively rare spinal vascular disease. It has gradually become more widely recognized in the past 30 years (1). Although it is a curable disease, delayed diagnosis is often associated with poor outcomes (2, 3). A large number of studies regarding the diagnosis and treatment of this disease are primarily published by neurosurgeons (3–9). Therefore, neurosurgeons may be relatively familiar with the diagnosis and treatment of this disease. However, the clinical manifestations of this disease in the early stage often include weakness and numbness of the bilateral lower limbs and abnormal gait, symptoms that are not specific to this disease and are very similar to the clinical manifestations of degenerative spine diseases. Patients are often first evaluated in orthopedics (10). Even when initial magnetic resonance imaging (MRI) findings of this disease are evident, misdiagnosis still occurs, and orthopedic surgery may even take place. According to the literature, the misdiagnosis rate for this disease is as high as 60%, and the clinical area with the highest misdiagnosis rate is orthopedics (10). As a result of misdiagnosis, the function of the spinal cord may be severely damaged or even permanently changed (11). Consequently, it is necessary to improve awareness of this disease among orthopedic surgeons. This article retrospectively analyzed the clinical data and prognosis of 22 patients with a SDAVF who were initially treated at an orthopedic clinic to identify the causes of misdiagnosis from the perspective of the orthopedic surgeon and to enhance the orthopedic surgeon's understanding of this disease.

## Methods

After approval was received from the Ethics Committee of our hospital, we retrospectively analyzed the data of the patients who were diagnosed *via* angiography and treated at the neurosurgery clinic of our hospital from January 1, 2013 to January 1, 2021. The patients were selected from those who were initially evaluated at the orthopedics clinic and were divided into two groups. Patients were included in group A (the correct diagnosis group) based on the following inclusion criteria: (1) diagnosis confirmed at the first visit by an orthopedic surgeon who recognized the disease; (2) diagnosis confirmed at the first visit by an orthopedic surgeon who did not recognize the disease but requested multidisciplinary teamwork (MDT) immediately, as a result of suspected diagnosis based on clinical or MRI findings, our MDT is a collaborative process conducted by multidisciplinary team of senior specialists, which is a discussion among doctors from different departments, including orthopedics, neurology, neurosurgery, urology, etc. Patients were included in group B (the misdiagnosis group) based on any of the following inclusion criteria: (1) no MRI or computed tomography (CT) examination was performed at the first visit, and treatment proceeded as if the patient had a spinal degenerative disease; (2) MRI findings of a manifested SDAVF that were treated as if they were spinal degenerative diseases because the orthopedic surgeon did not pay attention to the MRI findings.

The following variables were collected from medical charts and retrospectively assessed: general patient data, initial symptoms, diagnosed symptoms, initial imaging findings, causes of misdiagnosis and treatment results. SPSS 22.0 statistical software was used for statistical analysis. Quantitative data are expressed as the mean±SD, and the independent sample *t-*test was used for comparisons between the groups. The Fisher's exact test was used to compare qualitative data. *P* < 0.05 indicated that the difference was significant.

## Results

### General patient data

There were eight men and two women in group A. The ages ranged from 49 to 76 years (average age 61.24 ± 8.08). The average time from the onset of the disease to the first evaluation at the orthopedic clinic was 8.2 ± 1.4 months (range: 2–16 months). There were 10 men and two women in group B. Their average age was 62.37 ± 9.15 years (range: 51–78 years old). The average time from the onset of the disease to the first evaluation at the orthopedic clinic was 9.10 ± 0.9 months (range: 2–24 months). No significant differences were observed in the sex ratio (*P* = 0.63), age at onset (*P* = 0.66), and time to initial clinic evaluation (*P* = 0.08) between the two groups (Table 1).

**Table 1:** Comparison of patient data between the two groups.

**Group**	**Age**	**Ratio of males to females**	**Patients with typical MRI findings**	**Patients with concomitant degenerative spinal diseases**	**Time to initial clinical evaluation(m)**	**Time to confirmed diagnosis(m)**
Group A (10)	61.24 ± 8.08	8/2	8	1	8.2 ± 1.4	8.2 ± 1.4
Group B (12)	62.37 ± 9.15	10/2	3	7	9.10 ± 0.9	15.9 ± 1.6
*P*-value	0.66	0.632	0.015	0.026	0.08	<0.0001

### Initial symptoms and diagnosed symptoms

Of the 22 patients, three (14%) patients had limb numbness as the initial symptom, while four (18%) had motor weakness. The other 15 (68%) patients had both numbness and weakness as the primary symptom, six (27%) patients had accompanying low back pain and 10 (45%) patients had accompanying bladder and bowel dysfunction (both bladder and bowel incontinence in two patients, bladder incontinence in three patients and dysuria in five patients) (Table 2). Patients with lower limb weakness presented with spastic gait (6), neurogenic claudication (9), walking with a cane (3), and a wheelchair (1). Sensory disorders were mostly manifestations of atypical nerve root distribution. In addition, 10 patients (45%) had upper motor neuron (UMN) signs, five patients (23%) had lower motor neuron (LMN) signs, and seven patients (32%) had combined signs of UMN and LMN. In group B, there was a significant delayed diagnosis. Therefore, the symptoms of 12 patients in group B progressed to some extent at the time of diagnosis. Among these patients, case 2 was diagnosed with severe quadriplegia (Table 3).

**Table 2:** Initial clinical manifestations of 22 patients.

**Clinical manifestations**	**Number of cases**	**Constituent ratio (%)**
**Numbness or weakness**		
Numbness	3	14
Weakness	4	18
Numbness and weakness	15	68
Accompanying low back pain	6	27
Accompanying bladder and bowel dysfunction	10	45
Bladder and bowel dysfunction	2	9
Urinary incontinence	3	14
Dysuria	5	23

**Table 3:** Clinical characteristics of the included 22 patients.

**No**.	**Group**	**Initial symptoms**	**Diagnosed symptoms**	**Lesion level**	**MRI findings**	**Duration**	**Signs**	**Inclusion criteria for categorized group**
1	A	Numbness of lower limbs and perineal area, weakness,dysuria.	Same with the initial symptoms	Sacrococcygeal region	T2-hyperintense signals in the thoracic spinal cord,vascular flow empty in the spinal cord ventral and dorsal; vessels tortuous on the lumbar dural surface.	7	UMN and LMN	The orthopedic surgeon suspected a spinal DAVF, MDT consultation and made a definite diagnosis
2	A	Numbness and weakness of limbs, spastic gait	Same with the initial symptoms	C1	High cord T2 signal and flow voids in cervical spinal	10	UMN and LMN	The orthopedic surgeon confirmed the diagnosis
3	A	Weakness of lower limbs,dysuria,neurogenic claudication	Same with the initial symptoms	T8–9	High cord T2 signal and flow voids	2	UMN	The orthopedic surgeon confirmed the diagnosis
4	A	Numbness of lower limbs, weakness,dysuria	Same with the initial symptoms	T9–10	High cord T2 signal and flow voids	7.5	UMN	The orthopedic surgeon suspected a spinal DAVF, MDT consultation and made a definite diagnosis
5	A	Numbness of lower limbs,urinary incontinence	Same with the initial symptoms	T10–11	High cord T2 signal and flow voids	9	UMN	The orthopedic surgeon confirmed the diagnosis
6	A	Weakness of lower limbs, urinary retention	Same with the initial symptoms	T11–12	High cord T2 signal and flow voids	6.5	LMN	Orthopedic surgeon suspected a spinal DAVF, MDT consultation and made a definite diagnosis
7	A	Numbness of lower limbs, weakness,low back pain	Same with the initial symptoms	L1	High cord T2 signal and flow voids	5	LMN	The orthopedic surgeon suspected a spinal DAVF, MDT consultation and made a definite diagnosis
8	A	Numbness of lower limbs, weakness	Same with the initial symptoms	T8–9	High cord T2 signal and flow voids	9	UMN	The orthopedic surgeon suspected a spinal DAVF, MDT consultation and made a definite diagnosis
9	A	Numbness of lower limbs, weakness, low back pain, dysuria	Same with the initial symptoms	T11–12	High cord T2 signal and flow voids,spinal stenosis.	10	UMN and LMN	The orthopedic surgeon suspected a spinal DAVF, MDT consultation and made a definite diagnosis
10	A	Numbness of lower limbs, weakness, gatism	Same with the initial symptoms	T12	High T2 signal but no flow voids	16	LMN	Doesn't explain the symptoms,MDT consultation and made a definite diagnosis.
11	B	Weakness of right lower limb, low back pain, neurogenic claudication, dysuria	Numbness of lower limbs, weakness,walking with a cane	T10–11	High cord T2 signal and flow voids, Lumbar disc herniation	9	UMN and LMN	Misdiagnosed as lumbar disc herniation
12	B	Numbness of lower limbs, weakness, low back pain, urinary incontinence	Paraparesis, sensory disturbances, walking with a cane,urinary incontinence	T11	High cord T2 signal and flow voids, Lumbar disc herniation	16	LMN	Misdiagnosed as lumbar disc herniation
13	B	Numbness of lower limbs, weakness, low back pain, urinary incontinence	Paraparesis,urinary incontinence	L1–2	High cord T2 signal and flow voids, lumbar disc herniation	14	LMN	Misdiagnosed as lumbar disc herniation
14	B	Numbness of lower limbs, weakness,dysuria	Paraparesis,urinary incontinence	T11–12	High cord T2 signal and flow voids, lumbar spinal stenosis	17	UMN and LMN	Misdiagnosed as lumbar spinal stenosis and myelitis
15	B	Numbness of lower limbs, weakness,dysuria	Paraparesis,loss of sphincter control.	T9–10	High cord T2 signal and flow voids, lumbar spinal stenosis	19	UMN	Misdiagnosed as lumbar spinal stenosis and prostatic hyperplasia
16	B	Numbness of lower limbs, weakness, low back pain	Paraplegia,	T8–9	High cord T2 signal and flow voids, lumbar disc herniation	12	UMN	Misdiagnosed as lumbar disc herniation
17	B	Numbness of lower limbs, weakness	Paraplegia,dysuria	T6	High cord T2 signal and flow voids, lumbar spinal stenosis	11.5	UMN	Misdiagnosed as lumbar spinal stenosis and myelitis
18	B	Numbness of lower limbs, gatism	Severe paraparesis, loss of sphincter control	T10–11	High cord T2 signal and flow voids,lumbar spinal stenosis	17.5	UMN and LMN	Misdiagnosed as lumbar spinal stenosis and prostatic hyperplasia
19	B	Weakness of lower limbs, urinary retention	Numbness of lower limbs, weakness,dysuria	L1–2	High cord T2 signal and flow voids, lumbar spinal stenosis	14.5	UMN and LMN	Misdiagnosed as lumbar spinal stenosis and spinal cord tumo
20	B	Weakness of lower limbs, dysuria	Severe quadriplegia,loss of sphincter control.	T9	High cord T2 signal and flow void in the thoracic spinal cord, cervical stenosis, lumbar stenosis	28	UMN	Misdiagnosed as cervical spinal stenosis and lumbar spinal stenosis
21	B	Numbness of lower limbs, weakness	Paraplegia,dysuria	T9–10	No typical high T2 signal and flow voids	13.5	UMN	Misdiagnosed as thoracic spinal stenosis
22	B	Numbness of lower limbs, dysuria	Severe paraparesis,loss of sphincter control.	T10–11	High T2 signal but no flow voids	19	UMN	Misdiagnosed as thoracic spinal stenosis

### Imaging data

**Figure 1:**
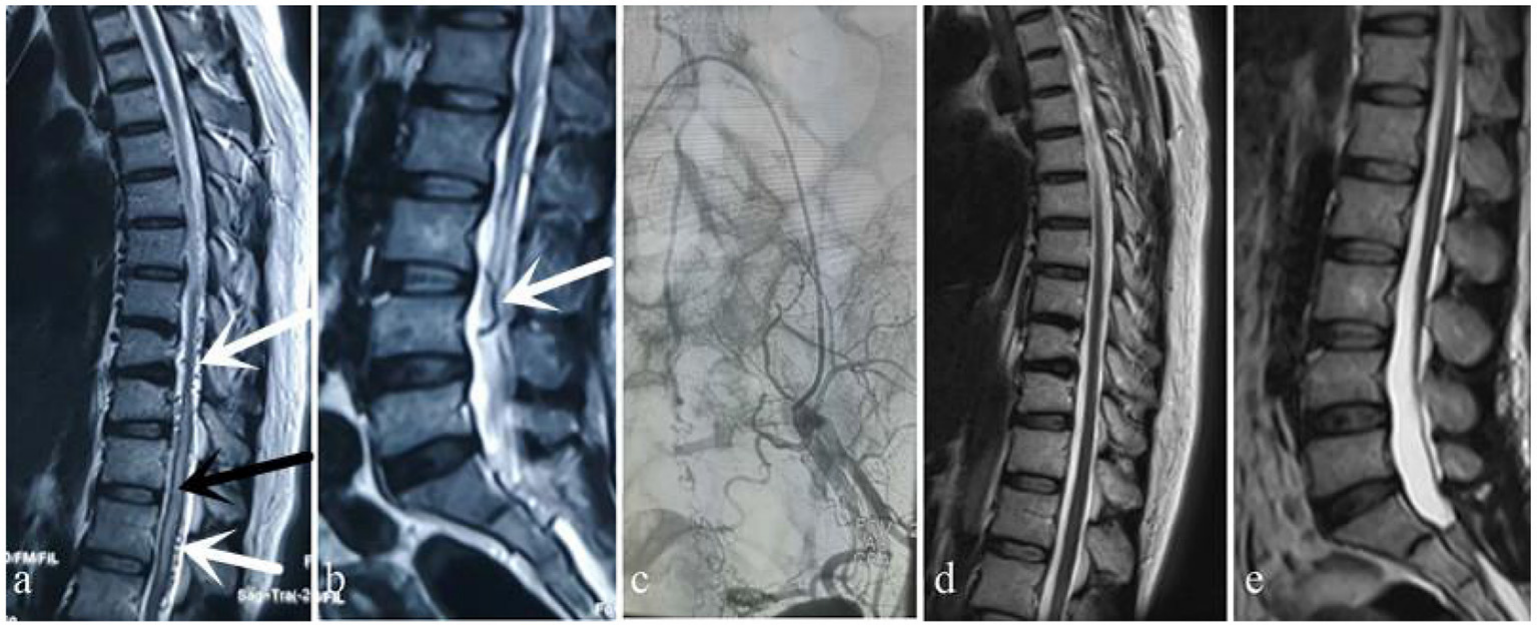
Case 1. A 49-year-old woman sought treatment 7 months after the onset of initial clinical symptoms of numbness and weakness of the bilateral lower limbs with urinary disturbance. The patient was first evaluated at the orthopedics clinic. An orthopedic surgeon suspected a spinal DAVF based on thoracic and lumbar MRI, which showed thoracic spinal cord edema and beaded changes on the dorsal side **(a)** (white arrow: “beaded” dilated flow void signals, black arrow: spinal cord edema) and tortuosity and dilation of the lumbar dural surface vessels **(b)** (white arrow: tortuous flow void signals). After MDT consultation, angiography performed by a neurosurgeon showed that the fistula was located in the sacrococcygeal region **(c)**. MRI 6 months after surgery showed that spinal cord edema and signs of flow voids had subsided **(d,e)**.

**Figure 2:**
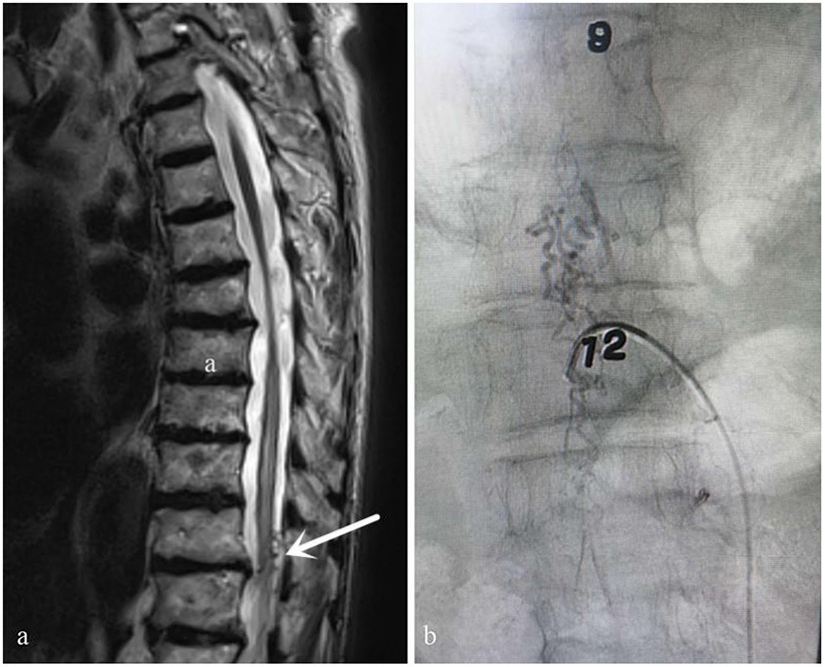
Case 2. A 73-year-old man who did not have typical signs of spinal cord edema and vessel flow voids on the cord on MRI **(a)** (white arrow: Atypical angiogram). This patient was misdiagnosed with thoracic spinal stenosis by an orthopedic surgeon. His symptoms were not completely consistent with the symptoms of thoracic spinal stenosis and worsened after he was treated for thoracic spinal stenosis. After MDT consultation, the diagnosis of spinal DAVF was confirmed *via* DSA performed by a neurosurgeon. The fistula was located at T9-10 **(b)**.

**Figure 3:**
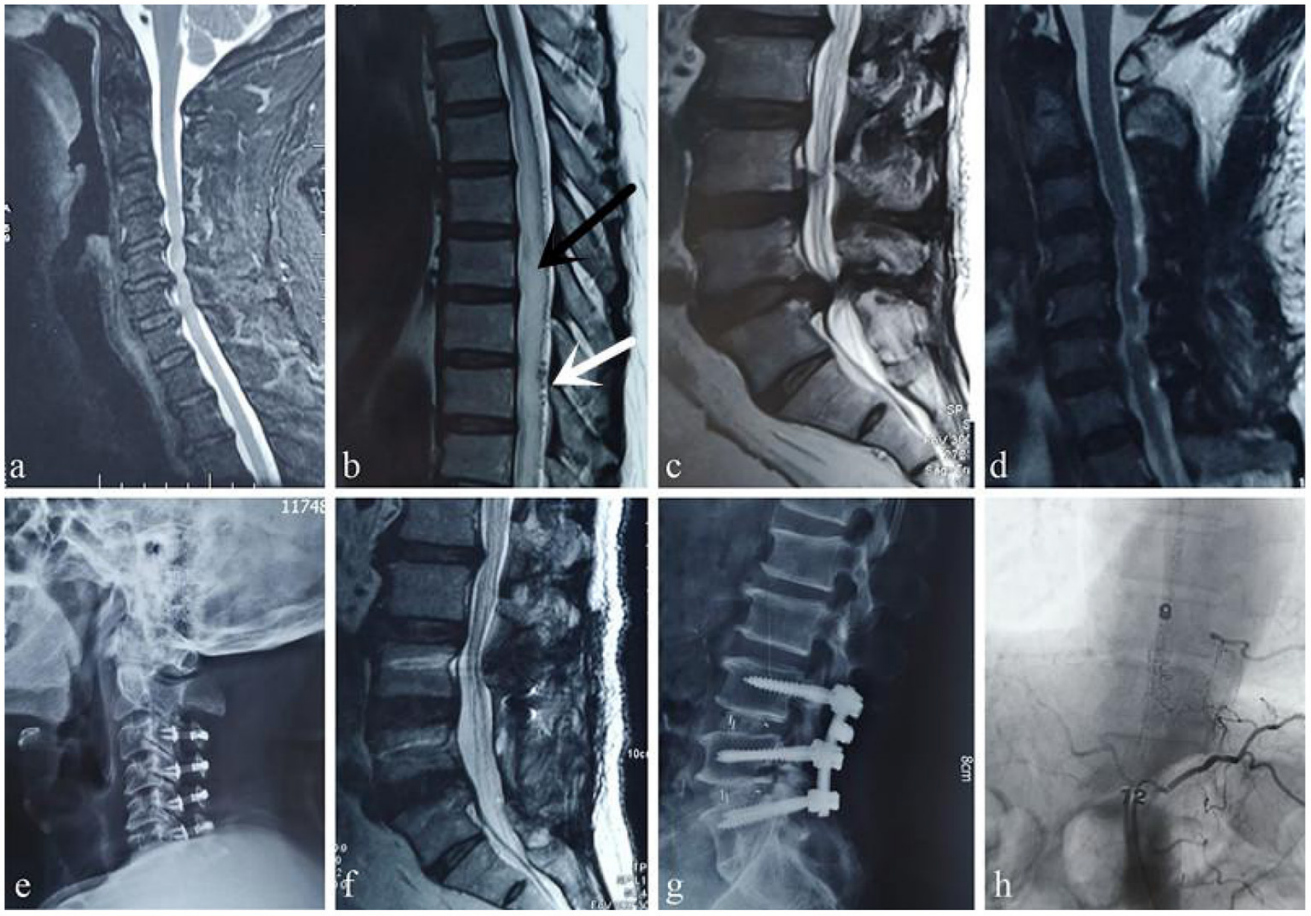
Case 3. A 59-year-old man was first evaluated at the orthopedic clinic because of “progressive lower extremity weakness and difficulty urinating for 2 month.” Cervical spine MRI shows spinal stenosis **(a)**, edema and signs of flow voids in the thoracic spinal cord, which are typical manifestations of a DVF **(b)** (white arrow: “beaded” dilated flow void signals, black arrow: spinal cord edema), and lumbar spinal canal stenosis **(c)**. However, the orthopedic surgeon ignored the diagnosis of a DVF; he first performed cervical spinal canal expansion and decompression **(d,e)** and then misdiagnosed the disease as cauda equina syndrome caused by lumbar spinal stenosis and performed lumbar spinal canal decompression **(f,g)**. These interventions aggravated the disease. The patient presented at our hospital approximately 9 months after the surgeries. After MDT consultation, angiography performed by a neurosurgeon confirmed that the fistula was at the T9 level **(h)**.

We defined MRI findings of T2-hyperintense signals in the spinal cord that were both edema of the spinal cord and “beaded” vascular flow empty in the spinal cord ventral and dorsal at the first clinic visit as a typical manifestation. In order to describe it more visually, some people refer to it as “white radish and black sesame” sign, where white radish refers to spinal edema signal (black arrow in Figures 1 and **3**), and black sesame refers to “beaded” vascular emptying signal (white arrow in Figures 1 and **3**). If the two above manifestations are absent or not obvious, we can consider it as an atypical MRI findings (Figure 2). In group A, those patients who were without spinal degenerative diseases, typical MRI manifestations were observed in eight patients, and one patient both had typical MRI findings and accompanied by spinal stenosis, MRI findings were atypical in 1 case. In group B, patients who were without spinal degenerative diseases, typical manifestations were observed in three patients, and seven patients had both typical MRI findings and degenerative diseases of the spine; two cases were atypical. Spinal digital subtraction angiography (DSA) revealed a SDAVF at lower thoracic spine (T5–10) for eight cases (Figure 3), thoracolumbar spine (T10–L2) for 10 cases (Figure 2), one case was located in sacrococcygeal segment (Figure 1), and one case was located in the cervical segment. The number of patients with typical MRI findings that without spinal degenerative disease was greater in group A than in group B (*P* = 0.015), while the number of patients with spinal degenerative diseases was significantly greater in group B than in group A (*P* = 0.026) (Table 1).

### Misdiagnosed disease and its treatment

In group A, three patients were diagnosed at the first evaluation at the orthopedics clinic because orthopedic surgeons recognized the disease and referred the patients to our hospital's neurosurgery clinics for further diagnosis. Six patients were diagnosed by neurosurgeons. Orthopedic surgeons did not recognize the disease in these six patients, but they found abnormally high T2 signals and vascular flow void signs of the spinal cord and initiated MDT consultation (Figure 1). An orthopedic surgeon believed that the existing spinal cord symptoms could not be interpreted in one patient and initiated MDT consultation. The patient was eventually diagnosed by MDT.

In group B, four patients reported low back pain. The orthopedic surgeon only ordered lumbar MRI at the first encounter and made a diagnosis of lumbar disc herniation. Of these patients, two were treated with steroids, which worsened the condition in a short period of time. These two patients were finally diagnosed with a SDAVF after MDT. One patient had an initial MRI of the thoracic spine that showed typical signs of a SDAVF and cervical and lumbar spinal stenosis. Considering that numbness and weakness were present in both upper limbs, cervical decompression was performed, but the lower limb symptoms and urinary dysfunction worsened after surgery. Orthopedic surgeons misdiagnosed the condition as lumbar spine cauda equina syndrome caused by lumbar canal stenosis and performed lumbar decompression. The patient's symptoms were more severely exacerbated after surgery. The orthopedic surgeon could not explain the cause of the aggravation. Finally, the patient was diagnosed by MDT (Figure 3). Two patients with atypical MRI findings were treated based on a misdiagnosis of thoracic spinal stenosis by an orthopedic surgeon based on pathological signs on physical examination (case 2). The remaining five patients were misdiagnosed with lumbar spinal stenosis and were treated with neurotrophic treatment, physical therapy or oral non-steroidal anti-inflammatory analgesics. These patients were misdiagnosed with myelitis, spinal cord tumor and prostate hyperplasia during consultation with a neurologist and urologist.

### Treatment effectiveness

All patients eventually underwent microsurgery treatment. The average follow-up duration was 11 months. In group A, the average time to confirmed diagnosis was 8.2 ± 1.4 months (range, 2–16 months). In group B, the average time to confirmed diagnosis was 15.9 ± 1.6 months (range: 2–24 months), and a significant difference was identified between the two groups (*t* = 12.04, *p* < 0.0001, Table 1). The average time to delayed diagnosis was 7 months in group B. The average follow-up period for both groups was 11 months (range: 0.5 to 1 year). At the last follow-up, The modified Aminoff-Logue Disability Scale scores showed statistically significant differences in terms of changes of the scores between preoperation and last follow-up in each group (*P* = 0.008 in group A and *P* = 0.021 in group B), scores between group A and group B at the last follow-up (*P* = 0.008), and improvement of the scores at the last follow-up between the two groups (*P* = 0.032) (Table 4).

**Table 4:** Aminoff–Logue scores before and after treatment.

**Group**	***n***	**Modified Aminoff–Logue myelopathy score**		
		**Preoperative**	**Last follow–up**	**Preoperative–last follow–up**
A	10	6.20 ± 2.20^a,c^	3.30 ± 2.16^a,d^	2.90 ± 1.20^e^
B	12	7.76 ± 2.23^b,c^	5.75 ± 1.71^b,d^	1.92 ± 0.79^e^

^*a,b,d,e*^, the P–values of the comparisons between the two groups were 0.008, 0.021, 0.008, and 0.032, respectively, (statistical significant). ^*c*^, the P–value of the comparison between the two groups was 0.116 (statistical significant).

## Discussion

### Initial symptoms and diagnosed symptoms

Our patients were predominantly men, accounting for 83.3% of all patients, and the diagnosis was most frequent in the early sixth decade, which is in agreement with previous series (12–16). SDAVF has an insidious onset in most patients and most of the initial symptoms lack specificity. The clinical manifestations mainly included limb numbness, weakness,and bladder and bowel dysfunction (4, 17). Rajanandini reviewed 153 patients with SDAVF,and the presenting symptoms included leg weakness (74 patients, 48.4%), leg sensory disturbances (41 patients, 26.8%), and sphincter disturbances (six patients, 3.9%) (18);Jellema K et al. reported that the most common symptoms were micturition problems (80%), leg weakness (78%), and numbness in the legs or buttocks (69%). The combination of all three symptoms was present in 58% of patients (7), higher than the 45% in our study. Other rare symptoms include back pain, and the symptom was back pain in 20% of our patients similar to other series (19, 20). Because this disease is prevalent in the thoracolumbar spinal cord, more than 80% of patients have fistulas located between the sixth thoracic vertebrae and the second lumbar spinal vertebrae (T6–L2) (21); In our research, the lesions were mainly located in the lower thoracic and thoracolumbar segments (81%). In group A, the lesion of 1 case located in C1, one case in the sacrococcygeal region, three cases in the lower thoracic vertebra (T5–10), and five cases in the thoracolumbar segment (T10–L2). In group B, the lesions of five cases located in the lower thoracic segment and seven cases in the thoracolumbar segment. There seems to be no obvious differences between group A and group B in regard to the lesion location(Table 3). Seventy –seven percentage of the patients (15) had UMN sign or the combined sign of UMN and LMN, which was higher than that reported in literature (18). The possible reason for this phenomenon might be that despite the lesions of some patients located in the lumbar segment, the thoracic spinal cord was also damaged due to the spread of spinal cord edema. in our study, most orthopedic surgeons treated the patients for lumbar degenerative diseases, and there seemed to be a lack of detailed physical examination of the patients in the clinic.

### Analysis of imaging findings

Spinal MRI is often the first choice of investigation. These images include (1) T2 hyperintense signals within the cord, (2) spinal cord expansion, and (3) vessel flow voids on the dorsal and/or ventral aspect of the cord. In chronic cases of SDAVF, there may be some spinal cord atrophy (22, 23). In our research, 19 patients (86%) had an area of T2 high signal intensity and flow void in the spinal cord, and the statistics are consistent with literature (18, 21, 23). There are also very few patients who do not have the above typical findings on MRI (24). As we demonstrated in case 2, there were no typical signs of spinal cord edema or vessel flow voids on the spinal cord on MRI. For these patients, DSA can be performed, and DSA is the gold standard.

In this study, although most orthopedic surgeons may not have been able to diagnose this disease initially, they had been able to identify the presence of abnormal MRI findings. However, in three patients who had typical MRI manifestations, orthopedic surgeons did not recognize the abnormal images, reflecting an ongoing lack of knowledge and awareness among treating physicians of this rare but serious disease (25). Misdiagnosis is most common in patients who have both venous fistulas and spinal degenerative diseases. Seven (58%) patients had been diagnosed with both diseases in group B, a proportion that was obviously higher than that in group A. One possible reason for misdiagnosis is that orthopedic surgeons often consider a diagnosis based on their own expertise and ignore the possibility of an arteriovenous fistula (case 3). Furthermore, in this study, four patients in the misdiagnosis group underwent MRI examinations that were limited to the lumbar spine. This approach may lead to misdiagnosis. Therefore, physical examination is indispensable for orthopedic patients. If symptoms representing upper motor neuron (UMN) signs are present, thoracic and cervical spine MRI should be performed (21).

### Analysis of treatment outcomes

The surgical procedure showed good results in terms of neurologic improvement (26–30). There is also a study that says that the improvement was non-significantly associated with younger age, acute onset, ambulant status and fistula below T9. However, in general, delays in the diagnosis and treatment of SDAVF appear to be associated with worse clinical outcomes for patients. In this study, the misdiagnosis group had poorer outcomes, which is consistent with the previous literature. Therefore, orthopedic surgeons who initially encounter patients with a SDAVF should have appropriate awareness of this disease. It should be emphasized that lumbar puncture, steroids or inappropriate surgery should be avoided until a SDAVF is completely excluded because these procedures can lead to rapid deterioration of neurological symptoms (2, 31, 32).

### Analysis of the causes of misdiagnosis

(1) SDAVF is a rare neurosurgical disease, with an annual incidence of only 5 to 10 cases per million (33), and some orthopedic surgeons lack adequate awareness of this disease. (2) The early symptoms for SDAVF patients are similar to those of degenerative spine diseases. Specialists often consider diagnoses based on their own expertise and ignore the possibility of a SDAVF diagnosis. (3) Orthopedic surgeons may have insufficient understanding of imaging findings, especially MRI images showing worm-like tortuosity and dilation of vessels. (4) If a patient has concomitant degenerative spine diseases, surgeons may quickly assume that the disease is strictly orthopedic disease and misdiagnose it. (5) The surgeon may not perform a careful physical examination or collect the patient's medical history, which may cause necessary imaging studies to be ignored, even when pathological signs are present.

### Countermeasures

In patients who present with limb numbness, weakness, bladder and bowel dysfunction, and upper motor neuron (UMN) signs present on physical examination, SDAVF is one of the diagnoses that should spring to mind; lumbar, thoracic and cervical spine MRI should be performed (21). If there are typical spinal cord edema and flow voids on MRI and degenerative spine diseases are excluded, a SDAVF can be diagnosed by DTM and DSA and can be treated by neurosurgery. If a SDAVF is associated with degenerative spine diseases, neurosurgery should be followed by orthopedic treatment.

If there is no typical spinal cord edema or flow voids on MRI, but there is evidence of degenerative spine diseases that can explain the present symptoms, a SDAVF can be treated by orthopedics; if there are no spinal edema and flow voids but there are degenerative spine diseases, then DTM is required or even DSA examination to exclude the possibility of a SDAVF.

**Figure 4:**
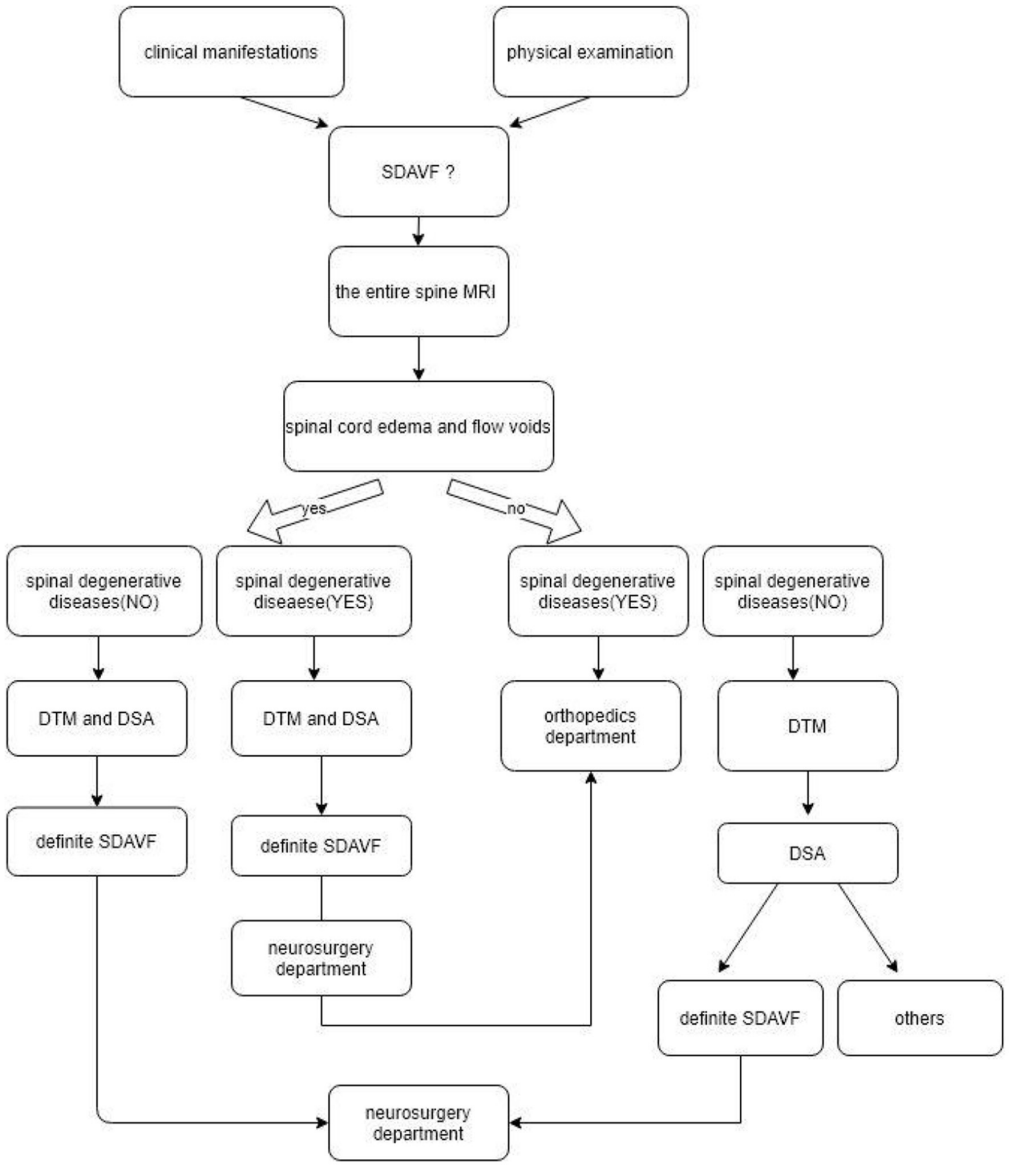
A detailed diagnosis and treatment flowchart for orthopedic surgeons.

A detailed diagnosis and treatment flowchart for orthopedic surgeons is shown in Figure 4.

However, in cases where MDT cannot be carried out in clinics, When common spinal stenosis fail to explain the symptoms,the possibility of spinal DAVFs should be considered. If lumbar MRI shows conus medullaris lesions, thoracic MRI should be performed, and recommended that the patients can be transferred to a higher medical institutions for further diagnosis.

### Limitations

This study has limitations; mainly, it is a single-center retrospective study with a small sample size and a short follow-up period. Although there were many limitations, we attempted to enhance the understanding of this disease among orthopedic physicians to improve diagnostic accuracy.

## Conclusion

Recommendations for orthopedic surgeons are as follows: In patients, especially those with limb numbness, weakness, and bladder and bowel dysfunction, diagnoses based exclusively on orthopedic expertise that ignore the possibility of a SDAVF diagnosis should not be considered. In addition to the routine observation of signs of spinal degeneration on MRI, attention should also be paid to signs such as spinal cord edema and flow voids. Timely MDT consultation is an important measure for reducing misdiagnosis, and lumbar puncture, steroids or inappropriate surgery should be avoided until a SDAVF is completely excluded.

## Data availability statement

The original contributions presented in the study are included in the article/supplementary material, further inquiries can be directed to the corresponding author/s.

## Ethics statement

This study was reviewed and approved by the Ethics Committee of the Second Affiliated Hospital of Xi'an Jiaotong University and complied with the guidelines outlined in the declaration of Helsinki. The patients/participants provided their written informed consent to participate in this study.

## Author contributions

BY designed the experiments. TL and XH performed the experiments. BY and XH collected and analyzed the data. TL and HL drafted manuscript. All authors contributed to the article and approved the submitted version.

## Conflict of interest

The authors declare that the research was conducted in the absence of any commercial or financial relationships that could be construed as a potential conflict of interest.

## Publisher's note

All claims expressed in this article are solely those of the authors and do not necessarily represent those of their affiliated organizations, or those of the publisher, the editors and the reviewers. Any product that may be evaluated in this article, or claim that may be made by its manufacturer, is not guaranteed or endorsed by the publisher.
